# Configuration Synthesis and Performance Analysis of Finger Soft Actuator

**DOI:** 10.1155/2018/4264560

**Published:** 2018-08-14

**Authors:** Ziqiang Zhang, Hanlong Chen, Zining Zhang

**Affiliations:** ^1^College of Mechanical Engineering and Applied Electronics Technology, Beijing University of Technology, Beijing 100124, China; ^2^School of Mechanical Engineering and Automation, Beihang University, Beijing 100191, China

## Abstract

Compared with the traditional rigid finger actuator, the soft actuator has the advantages of light weight and good compliance. This type of finger actuator can be used for data acquisition or finger rehabilitation training, and it has broad application prospects. The motion differences between the soft actuator and finger may cause extrusion deformation at the binding point, and the binding forces along nonfunctional direction may reduce drive efficiency. In order to reduce the negative deformation of soft structure and improve comfort, the configuration synthesis and performance analysis of the finger soft actuator were conducted for the present work. The configuration synthesis method for soft actuator was proposed based on the analysis of the physiological structure of the finger, and the soft actuator can make the human-machine closed-loop structure including *n* joints (*n* = 1, 2, 3) meet the requirement of DOF (degrees of freedom). Then the typical feasible configurations were enumerated. The different typical configurations were analyzed and compared based on the establishment of mathematical models and simulation analysis. Results show that the configuration design method is feasible. This study offers a theoretical basis for designing the configuration of finger soft actuator.

## 1. Introduction

Finger actuator can drive the fingers to move and can be used for data acquisition or finger rehabilitation training [1–6]. So it has broad application prospects. Considering that the rigid actuator have the disadvantages of poor compliance, cumbersome, and expensive, soft materials has been introduced into the robotic field [7–9]. Finger soft actuator has the advantages of softness and portability [10, 11]. Many researchers have conducted in-depth research on finger soft actuator [12–15].

Soft actuator is directly attached to the finger, and when the finger and the actuator are connected by a bandage, a spatial multiloop chain is formed. So the actuator and the finger constitute a typical man-machine integration system [16]. The soft actuator drives the finger to achieve movement through the physical interaction in the man-machine binding position. Therefore, if the motion of the soft actuator at the binding point does not match the trajectory of the finger, there is extrusion deformation at the binding point, and the binding forces along nonfunctional direction may reduce drive efficiency. There are two reasons that may affect the human-machine motion matching. The most important reason is that the soft actuator has the characteristics of continuous deformation, and it is different from the movement of the finger joints, which can be considered as revolute pairs. The second reason is that the axis of the finger joint cannot be accurately defined from the outside of the finger, which also leads to the movement deviation inevitably [17]. Although the soft actuator can realize flexible deformation under the action of external force, and this deformation can offset the movement difference, this will have a negative impact on comfort and driving efficiency. Therefore, it is an urgent problem, how to realize the match between human finger and machine without changing the advantage of soft actuator. To solve the above problems, the passive joints are introduced into the soft actuator, and the added DOF (degrees of freedom) ensure the actuator to be kinematically compatible with the finger of a human and hence avoid undesired interactional loads caused by mismatch of human finger and soft actuator. This idea was first proposed by Schiele and van der Helm [18]. A nine-DOF model of the human arm kinematics was presented and used to develop, test, and optimize the kinematic structure of a human arm interfacing exoskeleton, where two one-DOF passive joints were introduced in connecting position between human and exoskeleton forearms. Dehez and Sapin [19] presented a rehabilitation robot which was characterized by an action principle on the patient no longer requiring a tedious and accurate alignment of the robot and patient's joints. And the robot was connected to the arm through passive joints. Jarrassé and Morel [20] studied the general problem of connecting two similar kinematic chains through multiple passive mechanisms. Besides, many researchers have studied this problem deeply [21–24]. In addition to avoiding unnecessary force by increasing passive joints, some scholars have also studied how to achieve the trace of the axis by increasing the passive joints [25–28].

However, the objects of the above studies are rigid structure. Because there is a great difference for the movement mode between the soft structure and the rigid structure, the kinematic matching method for the soft actuator should be further analyzed. Besides, the above studies are aimed at number synthesis, and the effect of different passive joints on the movement performance is ignored. In particular, because the soft actuator has the characteristic of continuous deformation, it cannot achieve the axis tracking by adding the passive joints between the active joints.

In order to solve the above problems, the configuration design method is proposed in this paper according to the characteristics of continuous deformation of soft actuator to improve the comfort and driving efficiency, and analysis and comparison of typical configurations are conducted. Analysis and comparison results also prove the feasibility of the configuration design method. This study focuses on configuration synthesis for soft actuator and provides a reference for the configuration optimization and the mechanism analysis.

## 2. Methods

### 2.1. Physiological Structure of the Finger

**Figure 1:**
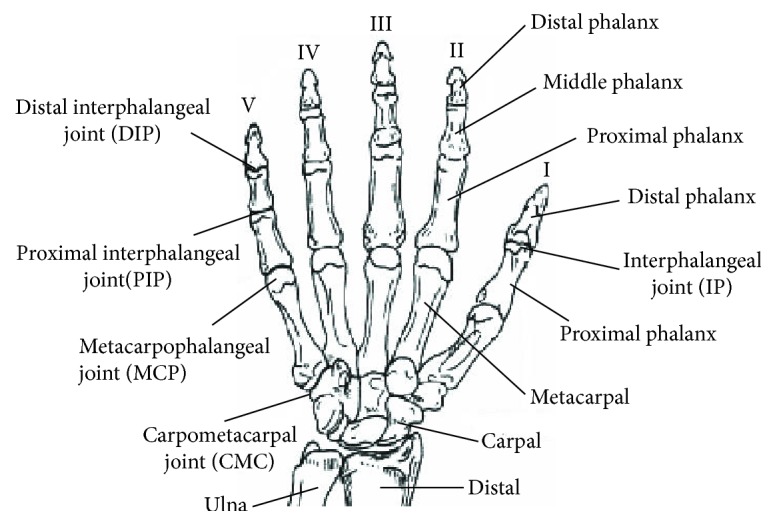
Diagram of the hand skeleton.

The hand is a complex locomotive organ with multiple joints. The structure of the hand is shown in Figure 1 [29]. The hand includes the thumb (I), the index finger (II), the middle finger (III), the ring finger (IV), and the little finger (V), and each finger consists of a metacarpal and three phalanges (the thumb has only two phalanges). The joints of the finger include the carpometacarpal joint (CMC), the metacarpophalangeal joint (MCP), and the interphalangeal joint. The CMC of the thumb is one of the most flexible joints and has two DOF. The motion ranges of CMC for fingers I–V are very small. MCP has one DOF for the thumb to make the thumb achieve flexion/extension movement, and it has two DOF for fingers II-V, which can make the fingers achieve flexion/extension movement and adduction/abduction movement. The interphalangeal joint has one DOF to make the fingers achieve flexion/extension movement. The thumb has only one interphalangeal joint (IP), and each one of fingers II–V has two interphalangeal joints: the proximal interphalangeal joint (PIP) and the distal interphalangeal joint (DIP).

**Figure 2:**
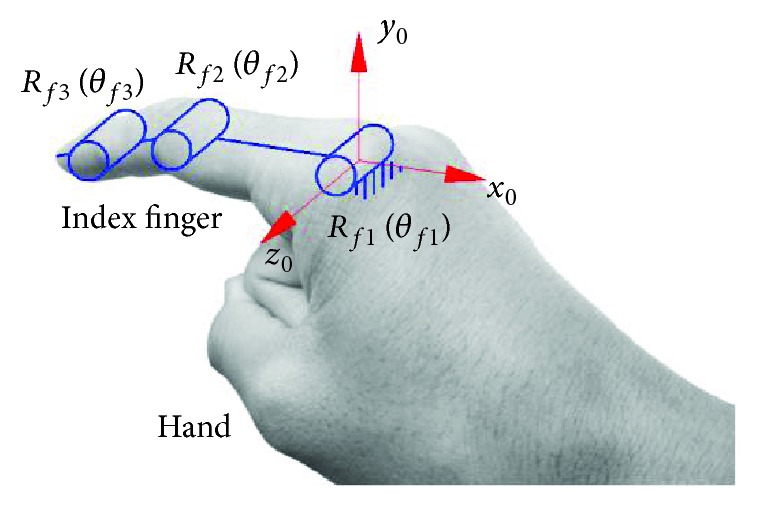
Equivalent model of the finger.

Take the index finger moving in a plane as an example. The metacarpophalangeal joint or the interphalangeal joint can be considered as one-DOF hinge. At this time, each finger has three DOF, and the equivalent model is shown in Figure 2. The axes of *R*_*fi*_ (*i* = 1, 2, 3) are parallel, and *θ*_*fi*_ (*i* = 1, 2, 3) is the relative rotation angle of two adjacent phalanges. The coordinate origin of the fixed coordinate system *o*_0_ − *x*_0_ *y*_0_ *z*_0_ coincides with the axis center of *R*_*f*1_. The direction of the *z*_0_-axis coincides with the axis of *R*_*f*1_, the direction of the *y*_0_-axis is upward, and the direction of the *x*_0_-axis can be determined according to the right-hand rule.

### 2.2. Soft Structure Design

**Figure 3:**
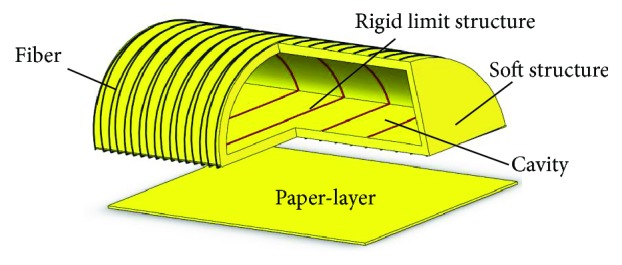
Schematic diagram of basic soft structure.

**Figure 4:**
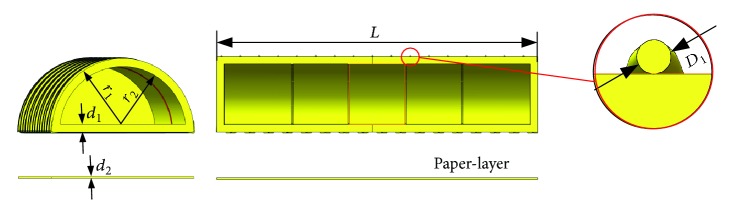
Schematic diagram of structural parameters.

The main factors which can affect the performance of the soft actuator are the material, driving mode, and structure. The material of the soft actuator is silicone rubber. The drive mode is pneumatic, which has the advantages of quick response and simple control. The basic structure of the soft actuator is shown in Figure 3, and it consists of main body structure, paper-layer, rigid fiber, and rigid limit structure. The cross section of the main body structure is a semicircle with cavity. Paper-layer is used to realize different motion modes. When the paper-layer is posted on the bottom of the structure, the soft structure realizes the bending deformation under air pressure because it limits the extension of the bottom. When the paper-layer is not used, the soft structure can realize the elongation deformation. Fiber is used to prevent the expansion of the soft structure in the cross section, which may reduce the amplitude of the bending or elongation. Rigid limit structure is installed on the inside of the cavity. It is used to prevent inward contraction of the bottom of the soft structure, which may lead to excessive expansion of the bottom, and result in the interference between the soft structure and the finger. The parameters of the soft structure are shown in Table 1 and Figure 4.

**Figure 5:**
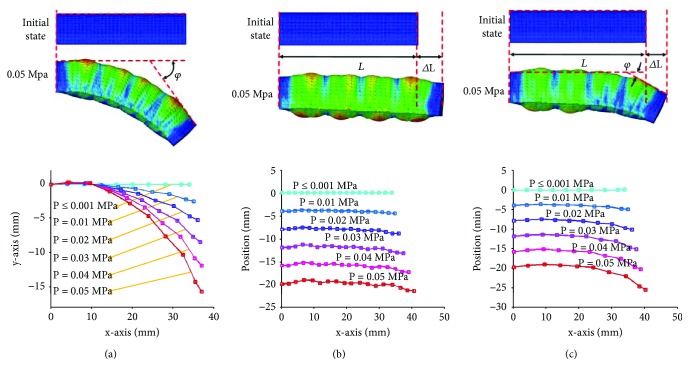
Deformation of soft structure. (a) Bending deformation; (b) elongation deformation; (c) compound deformation.

Different from the traditional rigid motion pair, the soft structure can be used alone or in combination. It means that the soft structure can not only realize the bending deformation or elongation deformation but also can realize compound motion through the irregular distribution of the paper-layer. For example, when the paper-layer is pasted on the bottom of half structure, soft structure can achieve both bending deformation and elongation deformation at the same time with only one driver. This is one of the important characteristics of the soft structure, and it enriches the motion form of the soft actuator. In order to prove the feasibility of soft structure design, the simulation is conducted with the ABAQUS. The material of soft structure is Elastosil M4601 silicone rubber. Neo-Hookean strain energy potential is defined by the coefficient *C* = 0.12 MPa. The material of fiber is elastic material with Young's modulus 31,067 MPa and Poisson's ratio 0.36. The material of rigid limit structure is number 45 steel.  Figure 5 shows the deformation process of the soft structure for the bending deformation, elongation deformation, and compound deformation. The design of the soft structure can meet the deformation requirement.

### 2.3. Configuration Synthesis of the Soft Actuator

**Figure 6:**
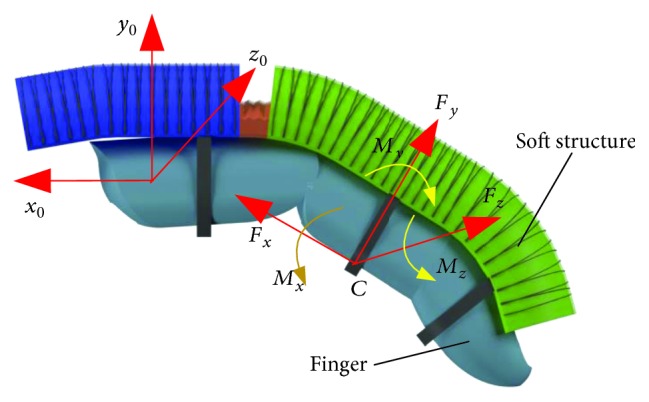
Schematic diagram of the binding forces.

The deformation of the soft structure is continuous, which is different from the rigid motion pair. The soft structure cannot correspond to the finger joint one by one, and the bionic design method cannot be used for the finger soft actuator. At this time, when the soft actuator and finger are fixed directly, movement deviation may make the finger being squeezed at the binding points and then lead to the finger suffering negative binding forces, such as friction force, pull force, or drag force. These negative forces and moments may have an impact on comfort and even cause injury to the fingers.  Figure 6 shows a situation in which the soft structure is directly connected with the finger. In binding point *C*, the finger may suffer the forces along three axes of moving coordinate system *o_c_*-*x_c_y_c_*, and it also may suffer the moments around three axes. Among the forces and moments, the force *F*_*y*_ and the moment *M*_*z*_ are the active driving force/moment, which can make the finger bending. Other forces and moments are negative forces (*F*_*x*_, *F*_*z*_)/moments (*M*_*x*_, *M*_*y*_). They cannot make the finger bend along the expected direction but will exert unnecessary pressure on the finger. Although negative binding forces can be offset by the material deformation of the soft structure, the superfluous deformation reduces the driving efficiency. So the configuration design method should be proposed to remove the negative binding forces between the soft actuator and the finger.

In order to carry out the configuration synthesis, the DOF of the soft structure should be determined first. Considering the soft structure is often under large deflection, which introduces geometric nonlinearities, the pseudo-rigid-body model is used in this study, which can simplify large-deflection analysis [30–32]. The soft structure can be replaced by *n* joints, and they are connected by equivalent links. The larger the number of *n* is, the more accurate the equivalent model is. The position of middle point of the *n*th equivalent link can be shown as

(1) $$\begin{array}{c} P_{1}=f_{1}\left(d_{1}\right),\\ P_{2}=f_{n-1}\left(d_{1},\theta_{1}\right),\ldots \\ P_{n-1}=f_{n-1}\left(d_{1},\theta_{1}\cdots \theta_{m}\cdots \theta_{n-1}\right),\\ P_{n}=f_{n}\left(d_{1},\theta_{1}\cdots \theta_{m}\cdots \theta_{n-1},\theta_{n}\right), \end{array}$$

where *d*_1_ is the elongation of the first equivalent prismatic pair which is used to equivalent replace the elongation deformation, and *θ*_*i*_ is the rotation angle of *n*th equivalent rotation pair, which is used to equivalent replace the bending deformation. In particular, the first equivalent joint also can be *θ*.

The cavity of the soft structure is subjected to distributed load in the motion process. Because the cavities are connected, it can be considered that the soft structure has only one driver. At this time, when the output pressure is determined, the deformation of the soft structure is also determined. So the air pressure and the shape of the soft structure are corresponding one by one. According to (1), the movement law of each joint is related to the motion law of the previous joints. When the first equivalent motion pair is determined, the attitudes of all other equivalent motion pairs are also determined. At this time, the soft structure can be seen as the *n* DOF mechanism with mutual coupling relationship.

**Figure 7:**
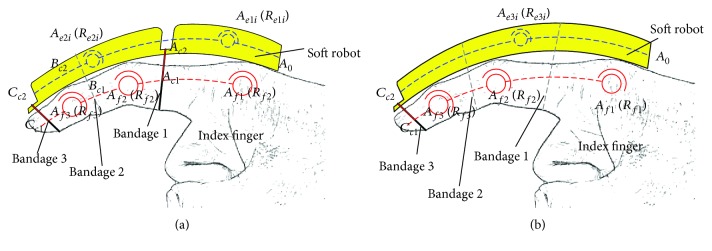
Different binding modes. (a) Driving for single joint and two joints, respectively; (b) driving for all joints simultaneously.

The finger soft actuator is a typical man-machine integrated system. Therefore, man-machine system should be studied instead of research on the soft actuator itself only. In general, the soft actuator is connected to the finger with a bandage directly. According to the number of finger joints that needs to be driven, there are three kinds of cases for the configuration synthesis of the soft actuator. (1) Case 1: driving for single joint: this means that the soft actuator should be able to drive a single joint independently. For example, in order to make the proximal phalanx rotate individually, the soft actuator and proximal phalanx are fixed by bandage 1, which is shown in Figure 7(a). *A*_*c*1_ is the binding position on the proximal phalanx, and *A*_*c*2_ is the binding position on the soft actuator. (2) Case 2: driving for two joints simultaneously: this means that the single-driver soft actuator can simultaneously drive two adjacent finger joints. At this time, the motion law of this two adjacent finger joints has a coupling relationship. For example, according to literature [33], the distal phalanx and the middle phalanx have a coupling relationship. It can be seen from Figure 7(a) that the soft actuator and the index finger are fixed at the distal phalanx by bandage 3, and there is no bandage between the soft actuator and the middle phalanx. *C*_*c*1_ is the binding position on the distal phalanx, and *C*_*c*2_ is the binding position on the soft actuator. In particular, bandage 2 which is between the soft actuator and the middle phalanx also can be added (the dotted line in Figure 7(a)). At this time, it can be considered as a combination of two single joint-driving modes, and the cavity of the soft structure is connected. (3) Case 3: driving for all the joints simultaneously: this means that the soft actuator can simultaneously drive all the finger joints. At this time, the soft actuator covers the whole finger, and the cavity of the soft actuator is connected. The soft actuator and the finger can be fixed only at the end of the distal phalanx (the solid line in Figure 7(b)) or in many different positions (the dotted line in Figure 7(b)), which also can be considered as a combination of multiple single joint-driving mode.

Because the palm and the end of the soft actuator can be considered as a frame, the soft actuator and the finger form multiple closed-loop structure. For case 1, *A*_*f*1_, *A*_*c*1_, *A*_*c*2_, and *A*_0_ form closed-loop 1, and it contains one finger joint *R*_*f*1_, where *A*_0_ is the frame of the soft actuator, and *A*_*f*1_ is the frame of the finger. In order to make the finger move without negative binding force, the DOF of closed loop should match the movement laws of the finger.

When the soft actuator and the finger are fixed directly, the motion screw can be shown as

(2) S1=001;000,S2=001;a1b10,S3=001;a2b20,S4=000;a3b30,…Sn+1=000;anbn0,

where *a* and *b* are real numbers, and they mean that the corresponding term is nonzero in motion screw. According to the screw theory, there are only three motion screws that are linearly independent for (2), and the reverse screw can be shown as

(3) S1r=000;100,S2r=000;010,S3r=001;000.

At this time, the DOF of closed-loop 1 is

(4) $$\begin{array}{c} M=d_{f}\left(m-g-1\right)+\overset{g}{\underset{i=1}{\sum }}f_{i}+\nu =3\left[\left(n+1\right)-\left(n+1\right)-1\right]+n+1=n-2, \end{array}$$

where *d*_*f*_ is the order of mechanism; *m* is the number of components; *g* is the number of motion pair; *f*_*i*_ is the DOF of *i*th motion pair; and *v* is the total number of over constraints. *n* is the number of equivalent joints of the soft actuator. At this time, the DOF is *n* − 2. Because the soft structure can be replaced by *n* driving joints, *M* should be *n*. So closed-loop 1 cannot move when the soft actuator is connected to the human finger directly without considering the extrusion deformation of the soft structure. In order to meet the requirements of the DOF of closed-loop 1, the passive joints should be added. The number of passive joints that are required is

(5) $$\begin{array}{c} t=n-3\left[\left(n+1\right)-\left(n+1\right)-1\right]-\left(n+1\right)=2. \end{array}$$

The passive joints can be rotation pairs or prismatic pairs. The motion screw of these motion pairs should be linearly independent. The axes of all the rotation pairs are parallel to the axis of the *R*_*f*1_, and all the prismatic pairs are perpendicular to the axis of the *R*_*f*1_. In particular, because there are coupling relations between *n* equivalent joints of the soft actuator, only one driving motor is needed to control the pseudo-rigid-body model, and the number of the DOF of the soft actuator also can be considered as one. When the soft actuator is connected to the human finger directly by bandage and two passive joints are added, the DOF of closed-loop 1 is

(6) $$\begin{array}{c} M=d\left(n-g-1\right)+\overset{g}{\underset{i=1}{\sum }}f_{i}+\nu =3\left(4-4-1\right)+4=1. \end{array}$$

This is consistent with the above analysis results.

For case 2, it can be known from Figure 7(a) that *A*_*c*1_, *C*_*c*1_, *C*_*c*2_, and *A*_*c*2_ form closed-loop 2, and it contains two finger joints *R*_*f*2_ and *R*_*f*3_. For closed-loop 2, both the distal phalanx and the middle phalanx can achieve flexion/extension movement. When the soft actuator is connected to the human finger directly by bandage, the DOF of closed-loop 2 is

(7) $$\begin{array}{c} M=d\left(m-g-1\right)+\overset{g}{\underset{i=1}{\sum }}f_{i}+\nu =3\left[\left(n+2\right)-\left(n+2\right)-1\right]+\left(n+2\right)=n-1. \end{array}$$

The DOF is not equal to *n*, and the passive joints are also needed. The number of passive joints that are required to be added is

(8) $$\begin{array}{c} t=n-3\left[\left(n+2\right)-\left(n+2\right)-1\right]-\left(n+2\right)=1. \end{array}$$

At this time, there is a coupling relationship for the distal interphalangeal joint and the proximal interphalangeal joint. When the soft actuator and the finger are tied at the distal phalanx and the middle phalanx, respectively, a closed-loop structure composed of the finger and the soft actuator can be seen as a series of two closed-loop 1. The DOF can be shown as

(9) $$\begin{array}{c} M=\overset{g}{\underset{i=1}{\sum }}f_{i}-dl=\left(n-m\right)+m+4+2-3\times 2=n. \end{array}$$

The DOF can meet the movement requirement. When the soft actuator and the finger are fixed only at the end of the distal phalanx, *A*_*f*1_, *C*_*c*1_, *C*_*c*2_, and *A*_0_ form closed-loop 3, and it contains three finger joints *R*_*f*1_, *R*_*f*2_, and *R*_*f*3_. The DOF of closed-loop 3 is

(10) $$\begin{array}{c} M=d\left(n-g-1\right)+\overset{g}{\underset{i=1}{\sum }}f_{i}+\nu =3\left[\left(n+3\right)-\left(n+3\right)-1\right]+\left(n+3\right)=n. \end{array}$$

At this time, the passive joints are not needed and there is a coupling relationship among three joints. However, because the soft actuator is only connected to the finger at the end of the distal phalanx, the joint axes of the finger and the soft actuator are easy to have deviation, especially the position which is far away from the bandage position. This deviation can make the equivalent rotation axes of the soft actuator not parallel to the finger joints. Take the soft structure with bending deformation as an example; when the soft structure is considered as a rotation pair, the motion screw of closed-loop 3 can be shown as

(11) S1=s1;0,S2=s1;r1×s1,S3=s1;r2×s1,S4=s2;r3×s2,

where **s** is the unit vector along the axis direction of the screw, and **r** is any point in the axis of the screw. The reciprocal screws are linearly independent, and the DOF of closed-loop 3 is −2. At this time, closed-loop 3 cannot move.

When the soft actuator and the finger are fixed at the distal phalanx, the middle phalanx, and the proximal phalanx, respectively, the closed-loop structure composed of the soft actuator and the finger can be considered as a series of closed-loop 1. The number of passive joints should meet the requirements of closed-loop 1. At this time, the DOF is

(12) $$\begin{array}{c} M=\overset{g}{\underset{i=1}{\sum }}f_{i}-dl=\left(n-m_{1}-m_{2}\right)+m_{1}+m_{2}+2\times 3+3-3\times 3=n. \end{array}$$

Similarly, for case 3, human-machine closed-loop also can be the combination of closed-loop 1 and closed-loop 2. For the space motion, such as the adduction/abduction movement of the metacarpophalangeal joint (PIP) being considered, the same configuration design method can be used.

The above study provides a way to relieve the negative binding forces between the soft actuator and the finger. This provides a reference for the design and selection of the configuration of the soft actuator.

## 3. Results

According to the above configuration design method, the closed-loop structure can be shown in Table 2. R_s_ and P_s_ mean the soft actuator with bending deformation and elongation deformation, respectively. (R_s_ *-*P_s_) means the soft structure with compound deformation. R_f_ means the equivalent finger joint. R and P are the passive joints. When the soft actuator drives the distal interphalangeal joint and the proximal interphalangeal joint simultaneously (case 2), the number of closed-loop structure can be 1 or 2. For the former, the soft actuator and the finger are fixed at the end of the distal phalanx. For the latter, the soft actuator and the finger are fixed at the end of the distal phalanx and the middle phalanx, respectively, and the closed loop can be considered as a series of two closed-loop 1. Similarly, when the soft actuator drives three joints simultaneously (case 3), the number of closed-loop structure can be 2 or 3. For the former, the soft actuator and the finger are fixed on the distal phalanx and the proximal phalanx, respectively, and it is the combination of closed-loop 1 and closed-loop 2. For the latter, the closed loop can be considered as a series of three closed-loop 1. For the different closed-loop structures, the order and position of motion pairs can be changed. In addition to one-DOF rotation pair and prismatic pair, other one-DOF joint also can be added in the closed loop, such as screw pair. However, other passive joints will increase the complexity of the structure. Considering the limitations of the structure, the closed loop with two prismatic pairs is not considered.

**Figure 8:**
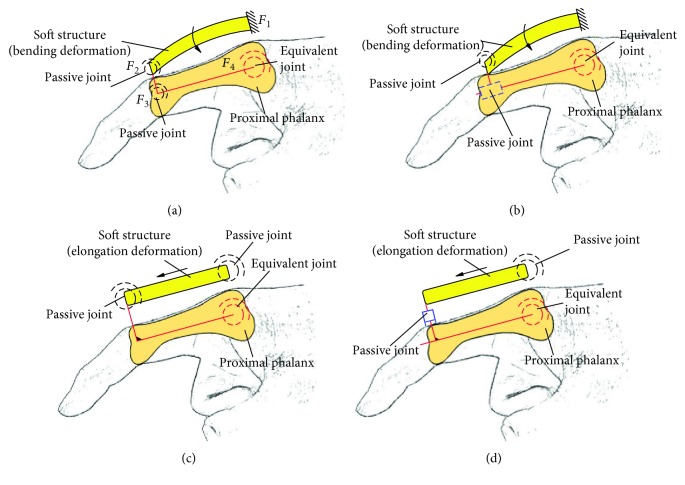
Typical structure forms for closed-loop 1. (a) 1R_s_-1R_f_-2R; (b) 1R_s_-1R_f_-1R1P; (c) 1P_s_-1R_f_-2R; (d) 1P_s_-1R_f_-1R1P.

Several typical structures for closed-loop 1 are shown in Figure 8. Figures 8(a)–8(d) correspond to configurations 1R_s_-1R_f_-2R, 1R_s_-1R_f_-1R1P, 1P_s_-1R_f_-2R, and 1P_s_-1R_f_-1R1P. The soft actuator with bending deformation and elongation deformation also can be replaced by compound deformation.

**Figure 9:**
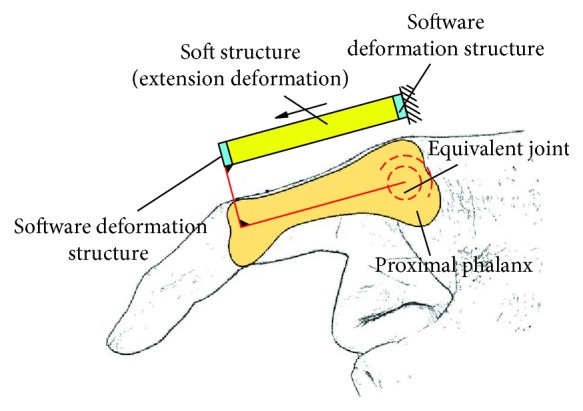
Schematic diagram for configuration 1P_s_-1R_f_-2R with soft equivalent passive joints.

In addition to the traditional design method for passive joint, the passive joint can also be realized by the deformation of the soft material to make the soft actuator be more simple. For example, the two passive rotation pairs of configuration 1P_s_-1R_f_-2R can be replaced by soft structures, and the schematic diagram is shown in Figure 9. At this time, the restoring force is inevitable, and it may affect the efficiency of the soft actuator and even limit the movement of the fingers. Therefore, comprehensive motion performance should be considered for selecting the appropriate passive joints.

**Figure 10:**
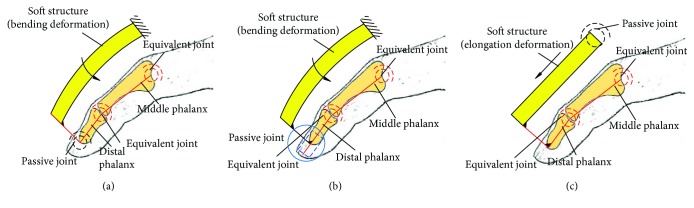
Typical structure forms for closed-loop 2. (a) 1R_s_-2R_f_-1R; (b) 1R_s_-2R_f_-1P; (c) 1P_s_-2R_f_-1R.

**Figure 11:**
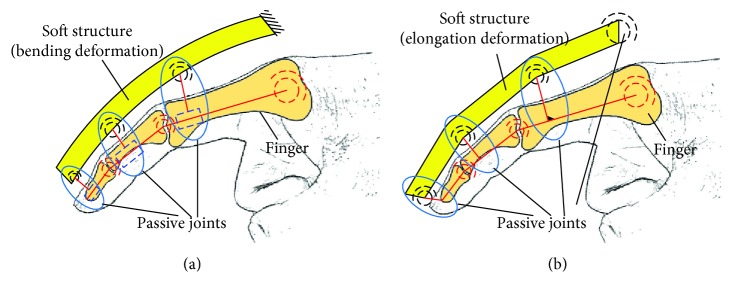
Typical structure forms for case 3. (a) 3-(1R_s_-1R_f_-1R1P); (b) 3-(1P_s_-1R_f_-2R).

Several typical structures for case 2 with one closed loop are shown in Figure 10. Figures 10(a)–10(c) correspond to configurations 1R_s_-2R_f_-1R, 1R_s_-2R_f_-1P, and 1P_s_-2R_f_-1R. In particular, case 2 with two closed loops is not listed, and the typical structures can be obtained according to Figure 8. The typical structures for case 3 with three closed loops are shown in Figure 11. Figures 11(a) and 11(b) show configuration 3-(1R_s_-1R_f_-1R1P) and 3-(1P_s_-1R_f_-2R), respectively. Similarly, the bending deformation and elongation deformation also can be replaced by compound deformation.

## 4. Discussion

In order to further verify the feasibility of the configuration synthesis, the motion performance analysis is needed. If the passive joints have large motion ranges, the necessity and feasibility of the configuration synthesis can be proved. It means that if there are no passive joints and the soft actuator and the finger are fixed directly, the movement of the passive joints will be offset by the extrusion deformation of the soft structure. And it may make the finger suffer the negative binding forces.

It can be known from Table 2 that there are many different configurations, and the configuration should meet the following principles. (1) The configuration should be feasible and simple. Reasonable closed-loop structure can make the mechanism avoid dead point and singular position in the movement process. (2) The soft actuator should not interfere with the fingers too much. And because the soft actuator will inevitably expand under air pressure, the actuator can easily interfere with the finger, and the interferences between the actuator and the finger with different configurations are different. (3) The soft actuator should have good force transmission characteristics. Force transmission characteristics includes two parts: the force of soft actuator on the finger and the force acting on a grasping object when the soft actuator and the finger are considered as a whole. For the former, the closed-loop structure should have high motion efficiency, which means that the deformation of the soft actuator can make the finger achieve flexible movement in large range. For the latter, the soft actuator should have large grasping force under the same pressure. Furthermore, since the soft actuator under the action of external forces is easy to deform, it should have good stability, which means that uncontrollable deformation of the soft actuator cannot occur.

### 4.1. Closed-Loop Structure Containing One Finger Joint (Case 1)

Take configurations 1R_s_-1R_f_-1R1P and 1P_s_-1R_f_-2R as examples. In order to analyze the effect of passive joints with material deformation, the configuration which is shown in Figure 9 is also analyzed.

**Figure 12:**
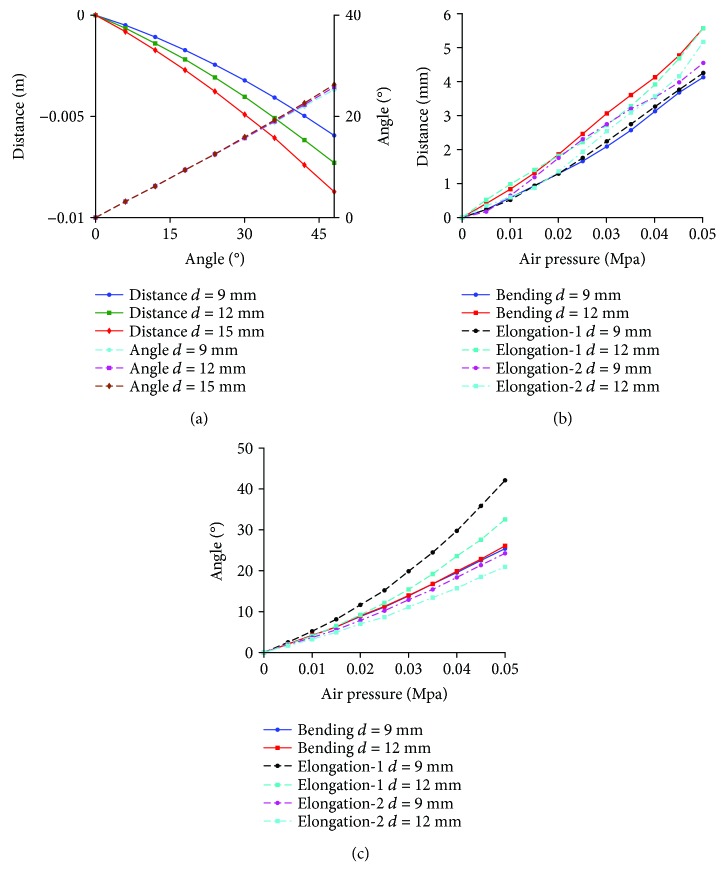
Movement law of the relative motion distance and the proximal phalanx. (a) Theoretical calculation results for configuration 1R_s_-1R_f_-1R1P; (b) simulation result for relative motion distance; (c) simulation result for rotation angles of the proximal phalanx.

**Figure 13:**
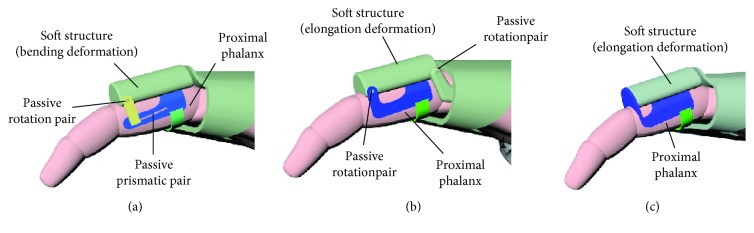
3D models of three typical configurations. (a) 1R_s_-1R_f_-1R1P; (b) 1P_s_-1R_f_-2R with traditional passive joints; (c) 1P_s_-1R_f_-2R with soft equivalent passive joints.

**Figure 14:**
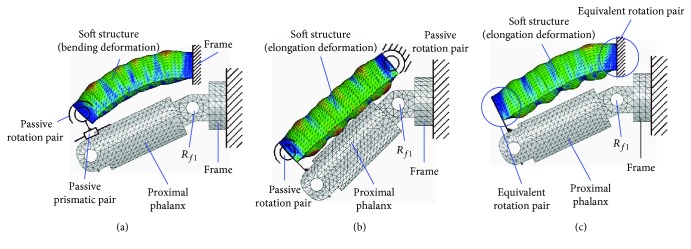
Motion states for three typical configurations for case 1. (a) Motion state for configuration 1R_s_-1R_f_-1R1P; (b) motion state for configuration 1P_s_-1R_f_-2R with traditional passive joints; (c) motion state for configuration 1P_s_-1R_f_-2R with soft equivalent passive joints.

**Figure 15:**
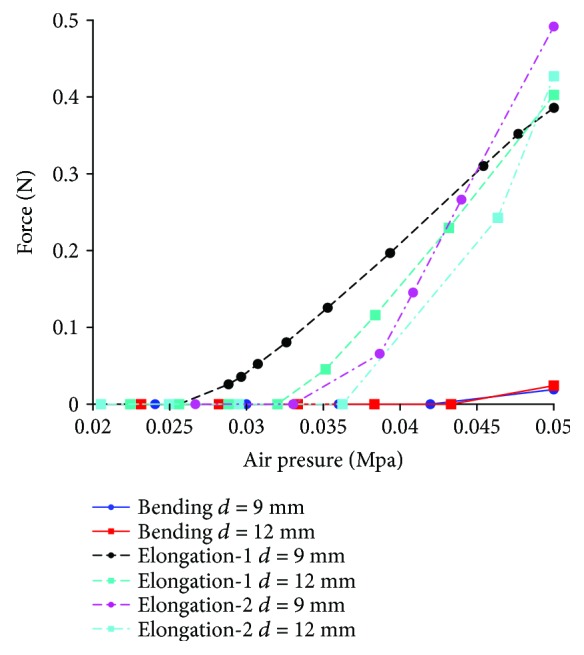
Change of grasping force with the range of air pressure (0 MPa, 0.05 MPa).

1. Relative motion of the soft actuator and the finger, suppose the length of the proximal phalanx is 44 mm: according to the theoretical analysis method (the theoretical analysis process is shown in Supplementary Materials (available here)), the moving distance of the slider (it reflects the relative movement between the soft actuator and the proximal phalanx) and the rotation angle of the proximal phalanx with different length of frame *d* for configuration 1R_s_-1R_f_-1R1P is shown in Figure 12(a). Negative value of vertical coordinate means the slider moves inward.
2. In order to further analyze the motion effect with different configurations, the simulation is conducted by the ABAQUS. Range of air pressure is (0 MPa, 0.05 MPa). Friction and the mass of the finger are no longer considered. Sectional dimensions of soft materials which are used for the passive joint are the same as the main body structure of the soft actuator, and the length is 1.5 mm. The relative motion is shown in Figure 12(b). “Bending” means the configuration 1R_s_-1R_f_-1R1P, and “elongation-1” and “elongation-2” mean the configuration 1P_s_-1R_f_-2R with traditional passive joints and soft passive joints, respectively. For the soft actuator with bending deformation, the relative motion is the sliding distance of the end point of the soft actuator on the finger. For the soft actuator with elongation deformation, the relative motion is the relative distance between the midpoint of the soft actuator and the corresponding point (pedal of the midpoint of the soft structure to the finger in the initial state) of the finger. Simulation result shows that the relative motion range between the soft actuator and the finger is large, and the maximum relative motion distance of three configurations has little difference. The rotation angle of the proximal phalanx under the same air pressure is shown in Figure 12(c). For different configurations, the proximal phalanx has large bending angle. Simulation result is consistent with the results of theoretical analysis.
3. The relative motion range and rotation angle of the proximal phalanx indirectly prove the feasibility of the configuration design. It means that if the passive joints are not added, the motion range of the passive joints needs to be offset by the extrusion deformation of the soft structure. This will lead to negative binding forces.
4. Structure complexity: different configurations correspond to different structural complexity. The 3D models of three typical configurations are shown in Figure 13. Due to limited position space, the design difficulty of the prismatic pair is greater than that of the rotation pair. So if the configuration 1R_s_-1R_f_-1R1P is used, the structure of the soft actuator is complex, and the structure of the soft actuator for configuration 1P_s_-1R_f_-2R with equivalent passive joints by material deformation is simple because no motion pairs are added.
5. Interference phenomenon: when the air pressure is 0.05 MPa, the motion states for three configurations are shown in Figure 14. The soft actuator and the proximal phalanx are not easy to interfere with the configuration 1R_s_-1R_f_-1R1P being used. The soft actuator is more likely to interfere with the proximal phalanx with the configuration 1P_s_-1R_f_-2R being used because of the expansion of the bottom and structural constraints. Compared with the configuration 1P_s_-1R_f_-2R with traditional passive joints, equivalent passive joints by material deformation can weaken the interference.
6. Force transmission characteristics: the simulation result for the change of grasping force is shown in Figure 15. The distance between the grasped object and the fixed coordinate system along the vertical direction is 15 mm, and the force along the vertical direction is measured. When the air pressure is 0.05 MPa, the grasping force corresponding to configuration 1R_s_-1R_f_-1R1P is much smaller than the grasping force corresponding to configuration 1P_s_-1R_f_-2R. The grasping force corresponding to configuration 1P_s_-1R_f_-2R with soft passive joints is the largest. Besides, it can be seen from the position where the grasping force begins to not be zero that the configuration 1P_s_-1R_f_-2R with traditional passive joints has a higher movement efficiency.

**Figure 16:**
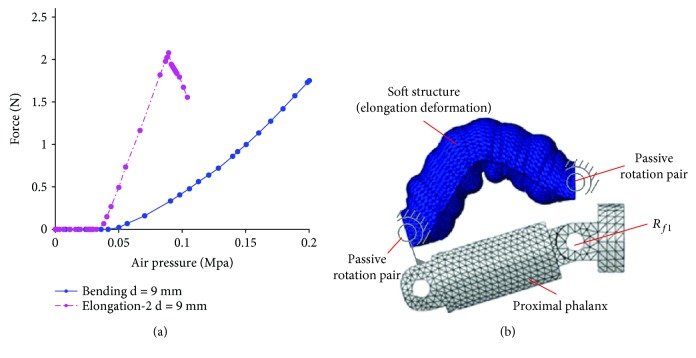
Instability phenomenon. (a) Change of grasping force with air pressure being larger 0.05 MPa; (b) simulation results about buckling deformation.

When air pressure continues to increase, the different configurations show new characteristics. Take configuration 1R_s_-1R_f_-1R1P and configuration 1P_s_-1R_f_-2R with soft passive joints as examples. It can be known from Figure 16(a) that when air pressure continues to increase, the grasping force with configuration 1R_s_-1R_f_-1R1P being used also increases. But the configuration 1P_s_-1R_f_-2R with soft passive joints is in a critical state of instability when the air pressure is 0.09 MPa. As air pressure continues to increase, the actuator becomes unstable. The simulation result is shown in Figure 16(b). Compared with configuration 1R_s_-1R_f_-1R1P, the soft actuator with elongation deformation as active driver is more likely to lose stability.

According to the above analysis results, the relative motion of the soft actuator and the finger demonstrates the necessity of configuration synthesis through the addition of passive joints. Structure complexity, interference phenomenon, and force transmission characteristics reflect the difference in the performance of different configurations. Performance comparison results for different configurations are shown in Table 3. In particular, Table 3 is only a relative comparison result.

### 4.2. Closed-Loop Structure Containing Two Finger Joints (Case 2)

**Figure 17:**
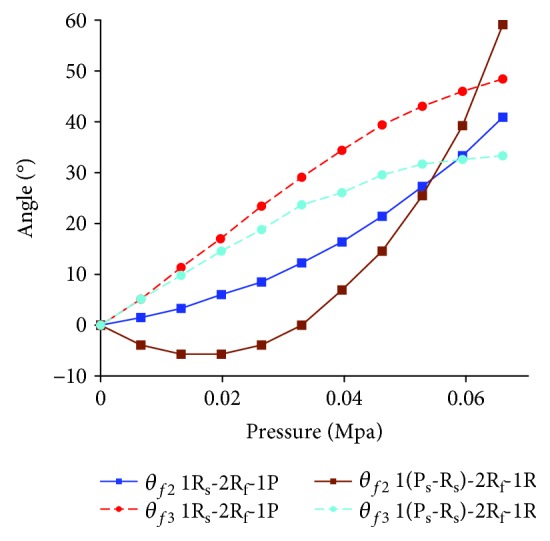
Simulation results about rotation angles of the middle phalanx and the distal phalanx for case 2.

**Figure 18:**
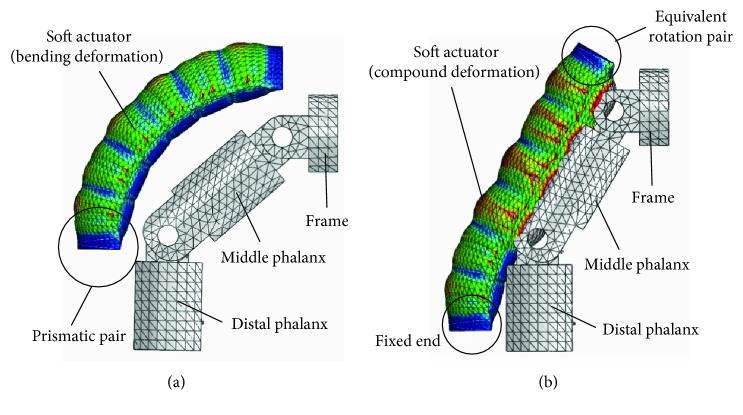
Motion states for two typical configurations for case 2. (a) Motion state for configuration 1R_s_-2R_f_-1P; (b) motion state for configuration 1(P_s_-R_s_)P_s_-1R_f_-2R with soft passive joint.

Take the configuration 1R_s_-2R_f_-1P and configuration 1(P_s_-R_s_)-2R_f_-1R with traditional passive joint as examples. Similarly, the simulation is conducted by the ABAQUS. The range of air pressure is (0 MPa, 0.066 MPa). The length of the frame is 9 mm. The movement laws of the middle phalanx and the distal phalanx for configurations 1R_s_-2R_f_-1P and 1(P_s_-R_s_)-2R_f_-1R are shown in Figure 17. When the air pressure is 0.066 MPa, the movement range of the middle phalanx and the distal phalanx is obvious. This reflects the necessity of adding the passive joints and feasibility of configuration design indirectly. In particular, the rotation angle of the middle phalanx of configuration 1(P_s_-R_s_)-2R_f_-1R with traditional passive joint first decreases and then increases, and negative value indicates that the middle phalanx has a tendency to reverse motion. The maximum reverse rotation angle is 5.8°. At this time, the material deformation can be used to replace the traditional passive joint. Besides, the limit mechanism also can be used to limit the rotation direction. The simulation result, with air pressure being 0.066 MPa, is shown in Figure 18. For configuration 1(P_s_-R_s_)-2R_f_-1R, when the traditional passive joint is replaced by soft passive joint, the distal phalanx does not have a reverse motion.

The above analysis results show that the configuration design method for case 2 (closed loop contains two finger joints) is feasible, and the compound deformation can enrich the configuration form.

### 4.3. Closed-Loop Structure Containing Three Finger Joints (Case 3)

**Figure 19:**
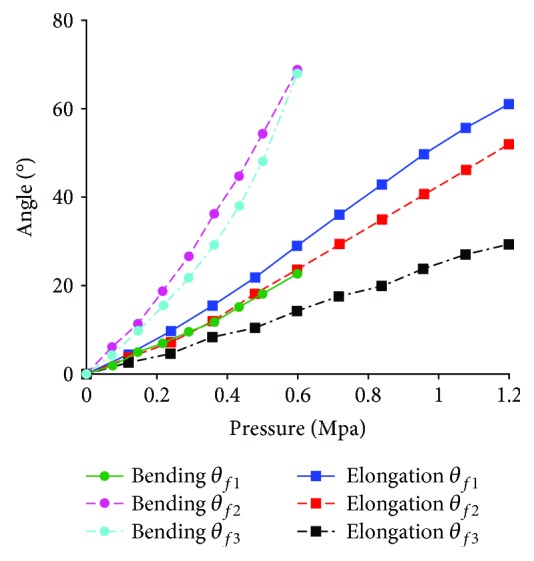
Simulation results about the rotation angles of three phalanges for case 3.

**Figure 20:**
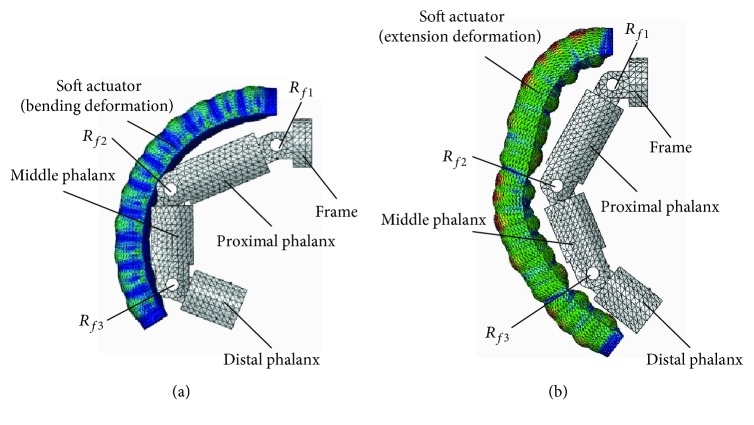
Motion states for two typical configurations for case 3. (a) Motion state for configuration 3-(1R_s_-2R_f_-1P); (b) motion state for configuration 3-(1P_s_-1R_f_-2R) with soft passive joints.

Take configuration 3-(1R_s_-1R_f_-1R1P) and configuration 3-(1P_s_-1R_f_-2R) as examples, and they are the series of three configuration 1R_s_-1R_f_-1R1P and three configuration 1P_s_-1R_f_-2R, respectively. The range of air pressure is (0 MPa, 0. 6 MPa) for configuration 3-(1R_s_-2R_f_-1P), and the range of air pressure is (0 MPa, 1.2 MPa) for configuration 3-(1P_s_-1R_f_-2R) with soft passive joints. The length of the frame is 9 mm.  Figure 19 shows the rotation angles of the proximal phalanx, the middle phalanx and the distal phalanx. The rotation angles of three phalanges for configuration 3-(1P_s_-2R_f_-1R) are smaller under the same pressure because the restoring forces generated by material deformation of the passive joints and the soft actuator in human-machine connection position inhibit the amount of deformation. The rotation angles of these three phalanges are obvious, and simulation results show that the configuration with series of multiple one-DOF closed-loop is feasible. Figures 20(a) and 20(b) show the end states for configuration 3-(1R_s_-2R_f_-1P) and 3-(1P_s_-1R_f_-2R) with soft passive joints, respectively. At this time, the interference phenomenon is more obvious for configuration 3-(1R_s_-2R_f_-1P) than that of configuration 3-(1P_s_-1R_f_-2R).

## 5. Conclusion

This paper focuses on the configuration synthesis of the finger soft actuator based on the motion difference between continuous deformation of the soft structure and joint movement of the finger to reduce the negative deformation of the soft structure and improve comfort. (1) The configuration synthesis method for finger soft actuator is proposed. This method can make the human-machine closed-loop meet the DOF requirement by adding the passive joints, and the man-machine negative binding forces can be relieved. The closed loop which contains one finger joint, two finger joints, and three finger joints and series of multiple closed loop are analyzed. (2) In order to further analyze the feasibility of human-machine closed-loop design method, the mathematical modeling and simulation are conducted for the closed loop containing different finger joints. The motion law of passive joints demonstrates the necessity of configuration design through the addition of passive joints. Otherwise, the movement of the passive joints will be offset by the extrusion deformation of the soft structure. And it may make the finger suffer the negative binding forces. (3) For the different configuration of case 1 (the closed loop contains one finger joint), the structure complexity, interference phenomenon, and force transmission characteristics are analyzed, and the analysis results reflect the performance difference of different configurations. The above research provides a reference for the design method and configuration optimization of the soft actuator. Further extensions to this work include structural parameter optimization, prototype design, and the experiment test.

## Figures and Tables

**Table 1:** Parameters of the soft structure.

*r*_1_ (outer radius)	*r*_2_ (inner radius)	*d*_1_ (thickness of bottom)	*L* (length)	*p* (pitch of rigid fiber)	*D*_1_ (section diameter of rigid fiber)	*d*_2_ (thickness of nondeformation layer)
16.0 mm	14.4 mm	0.7 mm	34.0 mm	2.0 mm	0.09 mm	0.5 mm

**Table 2:** Configuration of closed-loop structures.

Case	Number of closed-loop	Joint types
Case 1	1	1R_s_-1R_f_-2R, 1R_s_-1R_f_-1R1P, 1P_s_-1R_f_-2R, 1P_s_-1R_f_-1R1P, 1(R_s_-P_s_)-1R_f_-2R, 1(R_s_-P_s_)-1R_f_-1R1P
Case 2	1	1R_s_-2R_f_-1R, 1R_s_-2R_f_-1P, 1P_s_-2R_f_-1R, 1(R_s_-P_s_)-2R_f_-1R, 1(R_s_-P_s_)-2R_f_-1P
	2	2-(1R_s_-1R_f_-2R), 2-(1R_s_-1R_f_-1R1P), 2-(1P_s_-1R_f_-2R), 2-(1P_s_-1R_f_-1R1P), 2-[1(R_s_-P_s_)-1R_f_-2R], 2-[1(R_s_-P_s_)-1R_f_-1R1P]
Case 3	2	1(1R_s_-1R_f_-2R)1(1R_s_-2R_f_-1R), 1(1R_s_-1R_f_-2R)1(1R_s_-2R_f_-1P), 1(1R_s_-1R_f_-2R)1(1P_s_-2R_f_-1R)…
	3	3-(1R_s_-1R_f_-2R), 3-(1R_s_-1R_f_-1R1P), 3-(1P_s_-1R_f_-2R), 3-(1P_s_-1R_f_-1R1P), 3-[1(R_s_-P_s_)-1R_f_-2R], 3-[1(R_s_-P_s_)-1R_f_-1R1P]

**Table 3:** Comparison result of different configurations for closed-loop 1.

Configuration	Relative motion	Structure complexity	Interference	Mechanical property	Motion stability
1R_s_-1R_f_-1R1P	Little difference	Complex structure	Not easy to interfere	Mechanical property is not very good	Good stability
1P_s_-1R_f_-2R (traditional passive joints)		Complex structure	Prone to interference	Good mechanical property	Stability is not very good
1P_s_-1R_f_-2R (soft passive joints)		Simple structure	Prone to interference	Good mechanical property	Stability is not very good

## Data Availability

The data used to support the findings of this study are available from the corresponding author upon request.

## Supplementary Materials

Supplementary Materials The theoretical analysis method for the closed-loop structure containing one finger joint (case 1) is shown here, and the rotation angle of the finger and the movement laws of the passive joints can be obtained. This not only gives the mathematical modeling method of human-machine closed-loop but also provides reference for simulation results.
