# Supplementary material for: Configuration Synthesis and Performance Analysis of Finger Soft Actuator
Source: Appl Bionics Biomech. 2018 Aug 14;2018:4264560. doi: 10.1155/2018/4264560 (PMC6109997; doi:10.1155/2018/4264560)
Supplement: Supplementary Materials — The theoretical analysis method for the closed-loop structure containing one finger joint (case 1) is shown here, and the rotation angle of the finger and the movement laws of the passive joints can be obtained. This not only gives the mathematical modeling method of human-machine closed-loop but also provides reference for simulation results. [file 4264560.f1.pdf]

Establishment of a mathematical model

For the existing configurations (Table 2), take the configurations 1R$_s$-1R$_f$-1R1P and 1P$_s$-1R$_f$-2R as examples. In order to analyze the effect of passive joints with material deformation, the configuration which is shown in Fig. 9 is also analyzed.

The structure diagram of the configuration 1R$_s$-1R$_f$-1R1P, configuration 1P$_s$-1R$_f$-2R with traditional passive joints and configuration 1P$_s$-1R$_f$-2R with equivalent passive joints by material deformation are shown in Fig. 1s. According to pseudo-rigid-body model analysis method, the soft actuator with bending deformation is replaced by *n* rotation pair, and the soft actuator with elongation deformation is replaced by one prismatic pair. $A_{0i}A_{1i}$ (i=1, 2) is the frame. l_{fi} (i=1) is the length of each segment of finger, l_{eik} (i=1, ⋅⋅n) is the length of equivalent link of soft actuator. l_{si} (i=1, 2) is the length of link $A_{2i}A_{3i}$. The fixed coordinate system is the same as that in Fig. 2. The coordinate origin of moving coordinate system o_i-x_iy_i is in center of each pair, and the initial direction is the same as the fixed coordinate system.

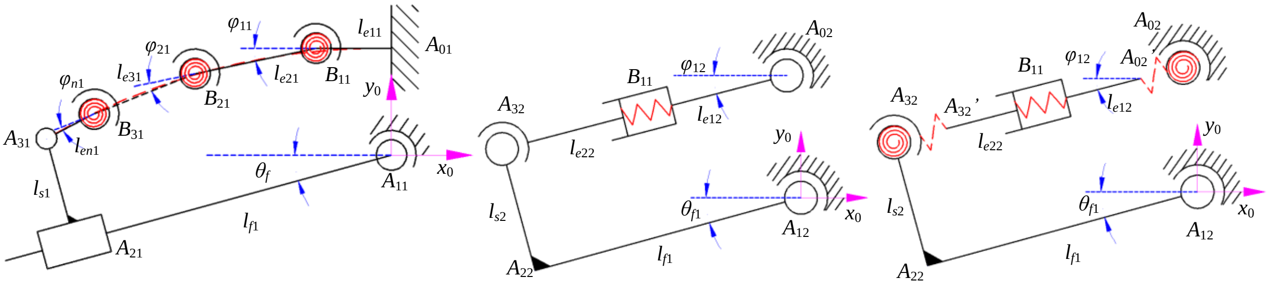

(a) (b) (c)

Figure 1s Schematic diagram for human-machine closed-loop 1. (a) Schematic diagram of configurations 1R$_s$-1R$_f$-1R1P; (b) Schematic diagram of configurations 1P$_s$-1R$_f$-2R with traditional equivalent passive joints; (c) Schematic diagram of configurations 1P$_s$-1R$_f$-2R with equivalent passive joints by material deformation.

Kinematic analysis is conducted first. For configuration 1R$_s$-1R$_f$-1R1P, the vector equation can be shown as

$$\boldsymbol{r}_{A01-A31}+\boldsymbol{r}_{A31-A21}+\boldsymbol{r}_{A21-A11}+\boldsymbol{r}_{A11-A01}=\boldsymbol{0} \tag{1s}$$

where, $\boldsymbol{r}$ is the position vector. According to the position relationship, eq. (1s) can be written as

$$\begin{cases} l_{e11}+\sum_{i=2}^{n+1}\left(l_{ei1}\cdot\cos\sum_{j=1}^{i-1}\varphi_{j1}\right)=l_{f1}\cdot\cos\theta_{f1}+l_{s1}\cdot\sin\theta_{f1} \\ \sum_{i=2}^{n+1}\left(l_{ei1}\cdot\sin\sum_{j=1}^{i-1}\varphi_{j1}\right)=l_{f1}\cdot\sin\theta_{f1}-l_{s1}\cdot\cos\theta_{f1}+d \end{cases} \tag{2s}$$

where, φ_{j1} is rotation angle of equivalent rotation pair of soft actuator, and θ_{f1} is rotation angle of proximal phalanx. In eq. (2s), in addition to the unknown number θ_{f1} and displacement of sliding block, φ_{j1} also should be determined. Because the bending deformation of the soft structure is caused by the air pressure acting on the cross section, so the bending process can be considered that the end of the soft actuator is subjected to moment. The moment (moving coordinate system in each equivalent motion pair of soft actuator) can be written as

$$\varphi_{ji}\cdot k_{i1}=M_i \tag{3s}$$

where, k_{i1} is the equivalent spring coefficient. The rotation angle of the end of soft actuator can be shown as

$$\varphi_m=\sum_j\varphi_{j1}=M_{eb}\left[1,1,\cdots,1\right]\begin{bmatrix}1/k_{21}\\1/k_{31}\\\vdots\\1/k_{i1}\end{bmatrix} \tag{4s}$$

where, M_{eb} is equal to the moment generated by the expansion of the upper surface of the soft structure under air pressure, and it also equal to the moment generated by the equivalent torsion springs. The displacement of the end of the soft actuator can be expressed as

$$s_m = \sum_j \left(\varphi_{j1} \bullet \sum_{l=i}^{n} l_{el1} \right) = M_{eb} \left[\sum_{i=2}^{n} l_{ei1}, \sum_{i=3}^{n} l_{ei1}, \cdots, \sum_{i=n}^{n} l_{ei1} \right] \begin{bmatrix} 1/k_{21} \\ 1/k_{31} \\ \vdots \\ 1/k_{i1} \end{bmatrix} \tag{5s}$$

Then k_{i1} can be determined by optimization method. Optimization objective function can be written as

$$J = \sum_{i=1}^{n} \rho_i \frac{\left(a_{ri} - a_{mi}\right)^2}{a_{ri}^{\ 2}} \tag{6s}$$

where, a_{ri} is the motion parameters for soft actuator, and a_{mi} is the motion parameters for equivalent series structure. ρ_i is the proportional parameter to determine the weights of different performance parameters. According to the above method, φ_{j1}, displacement of sliding-block and θ_{f1} can be obtained.

In order to analyze the mechanical properties, the force analysis should be conducted. Considering the soft actuator is replaced by n rotation pairs with torsion springs, the links can be subjected the moments produced by torsion springs. At this time, The force and moment balance equation can be written as

$$\begin{cases} \sum_{i=1}^{n} \boldsymbol{F}_i + \sum_{i=1}^{n} \boldsymbol{f}_{ti} + \boldsymbol{f}_{ci} + m_i \boldsymbol{g} = \boldsymbol{0} \\ \sum_{i=1}^{n} \left(\boldsymbol{r}_{Fi} \times \boldsymbol{F}_i \right) + \sum_{i=1}^{n} \left(\boldsymbol{r}_{fti} \times \boldsymbol{f}_{ti} \right) + \boldsymbol{r}_{mi} \times \left(\boldsymbol{f}_{ci} + m_i \boldsymbol{g} \right) + \sum_{i=1}^{n} \boldsymbol{M}_i + \sum_{i=1}^{n} \boldsymbol{m}_{ti} + \boldsymbol{M}_{ci} = \boldsymbol{0} \end{cases} \tag{7s}$$

where, $\boldsymbol{F}_i$ is internal force of each joint, $\boldsymbol{f}_{ti}$ is external force, and m_i is the mass of each link. $\boldsymbol{r}$ ($\boldsymbol{r}_{Fi}$, $\boldsymbol{r}_{fti}$ and $\boldsymbol{r}_{mi}$) is the position vector of each force, and it is the function of θ_{f1} or φ_{j1}. $\boldsymbol{M}_i$ is the internal moment, and $\boldsymbol{m}_{ti}$ is the external moment. For the equivalent link of pseudo-rigid-body model, $\boldsymbol{M}_i$ means the torque generated by torsion springs. For finger joint and passive joint with rotation pair, $\boldsymbol{M}_i$ is zero. $\boldsymbol{f}_{ci}$ and $\boldsymbol{M}_{ci}$ is the inertia force and inertia moment.

Considering the attitude of soft actuator is unique in the case of air pressure unchanged, for simplicity, the kinematic analysis of the soft actuator also can be carried out by the position constraint. For the pseudo-rigid-body model, the reference point is the middle point of the n-th link, and the position points can be obtained by experiment.

For configuration $1P_s$-$1R_f$-2R with traditional passive joint, the position vector equation can be shown as

$$\boldsymbol{r}_{A02-A32} + \boldsymbol{r}_{A32-A22} + \boldsymbol{r}_{A22-A12} + \boldsymbol{r}_{A12-A02} = \boldsymbol{0} \tag{8s}$$

Because the prismatic pair is active motion pair, so the eq. (8s) can be simplified as

$$\left(l_{e12} + l_{e22}\right)^2 = \left(l_{f1} \cos\theta_{f1} + l_{s2} \sin\theta_{f1}\right)^2 + \left(l_{f1} \sin\theta_{f1} + d - l_{s2} \cos\theta_{f1}\right)^2 \tag{9s}$$

In order to solve θ_{f1}, suppose x=tan(θ_{f1}/2), so the trigonometric function can be shown as

$$\sin\theta_{f1} = \frac{x}{1+x^2}, \quad \cos\theta_{f1} = \frac{1-x^2}{1+x^2} \tag{10s}$$

Inserting eq. (10s) into eq. (9s), eq. (9s) can be simplified as one element four times equation. Then θ_{f1} can be obtained. In particular, for the soft actuator with elongation deformation, it can only move along the the vertical direction of the cross section, so the equivalent force is

$$F_{ee} = k_{ee} \cdot l_{ee} \tag{11s}$$

where, k_{ee} is the equivalent spring coefficient, and l_{ee} is elongation. The equivalent force F_{ee} acting at the end of the soft actuator also can be written as

$$F_{ee} = \frac{1}{2}\left(P_1 - P_{atm}\right)\cdot \pi \cdot {r_2}^2 \tag{12s}$$

where, (P_1-P_{atm}) is the air pressure of the soft structure suffers. k_{ee} can be determined according to eqs. (11s)-(12s), and the force analysis process can be obtained by eq. (7s).

For configuration $1P_s$-$1R_f$-2R with equivalent passive joints by material deformation, the material deformation can be expressed according to eq. (1). The torsion springs are used in rotation pairs and the linear springs are used in prismatic pairs to replace the elastic restoring force of the material. Considering the length of soft material which is used to replace the passive joints is very short, only one torsion springs are used in $A_{02}A_{02}'$ and $A_{32}A_{32}'$. At this time, kinematic equation is the same as eq. (8s). Force balance equation and moment balance equation are the same as eq. (7s).
